# Does the speaking valve improve tracheostomized ICU patients communication and emotional security?

**DOI:** 10.1186/2197-425X-3-S1-A557

**Published:** 2015-10-01

**Authors:** O Vallès Fructuoso, B Ruiz de Pablo, M Fernández Plaza, MJ Fernández Olgoso, G Martínez Estalella

**Affiliations:** Intensive Cares, Hospital Universitari de Bellvitge, Hospitalet de Llobregat, Spain; Research and Education, Hospital Universitari de Bellvitge, Hospitalet de Llobregat, Spain; Clinical Nursing, Universitat de Barcelona, Hospitalet de Llobregat, Spain

## Introduction

Speaking is our best way of communication, losing voice after a tracheostomy affects seriously patients way of life. With the speaking valve they could have the opportunity of speaking and communicating without using other ways of no oral communication. It could be used as soon as the patient could breathe by his own.

## Objectives

The aim of this study is to evaluate the quality of communication in tracheostomized ICU patients during weaning period and to assess the importance placed on the ICU patient security related to communication barriers.

## Methods

Experimental pilot study. The sample includes 10 tracheostomized patients admitted in a long stay intensive care unit. They should be on late-stage weaning (more than 1 day) and have a < 2 degree of dysphagia maintaining the cuff deflated. Patients with neurological involvement in expression and communication were excluded. We assessed the communication degree pre- and post-using the speaking valve through the SPEACS-2 scale. To assess the emotional and social impact on patient safety we used the screening questionnaire of emotional distress. The data analysis was performed using the statistical program R Commander.

## Results

The average time patients carrying tracheostomy is 21 days with a mean stay in ICU for 40 days. The 80% had emotional distress due to emotional disorders (60%), lack of family contact (40%) and the fear of not speaking again (30%). The 40% had difficult communication. Only 30% of patients do not tolerate a session of 30 minutes with the speaking valve because of desaturation below 85% and abundant secretions. An improving emotional distress has been observed in 100% of patients after using the speaking valve.

## Conclusions

There is an ongoing emotional distress in tracheostomy critically ill long lasting patients. The fear never to speak again and difficulty communicating with family, staff and the communication of adverse effects are the main reasons. Using the speaking valve disappears emotional distress and they feel more confident in being able to communicate in an effective way both to the staff as to the family.They feel more confident when communicating their needs, fears or concerns.

## Grant Acknowledgment

Many thanks to Dr. Diaz Prieto and Gemma Castañeda Fernández for their help and support.
